# Antibacterial efficacy of novel bismuth-silver nanoparticles synthesis on *Staphylococcus aureus* and *Escherichia coli* infection models

**DOI:** 10.3389/fmicb.2024.1376669

**Published:** 2024-04-08

**Authors:** Beatriz Elena Castro-Valenzuela, Moisés Armides Franco-Molina, Diana Ginette Zárate-Triviño, Licet Villarreal-Treviño, Jorge R. Kawas, Paola Leonor García-Coronado, Gustavo Sobrevilla-Hernández, Cristina Rodríguez-Padilla

**Affiliations:** ^1^Laboratorio de Inmunología y Virología, Facultad de Ciencias Biológicas, Universidad Autónoma de Nuevo León, San Nicolás de los Garza, Nuevo León, Mexico; ^2^Posgrado en Microbiología, Facultad de Ciencias Biológicas, Universidad Autónoma de Nuevo León, San Nicolás de los Garza, Nuevo León, Mexico; ^3^Posgrado Conjunto Agronomía-Veterinaria, Universidad Autónoma de Nuevo León, General Escobedo, Nuevo León, Mexico

**Keywords:** antibacterial, nanoparticles, bismuth, silver, bimetallic, infection, *Staphylococcus aureus*, *Escherichia coli*

## Abstract

**Introduction:**

The emergence of multi-drug-resistant bacteria is one of the main concerns in the health sector worldwide. The conventional strategies for treatment and prophylaxis against microbial infections include the use of antibiotics. However, these drugs are failing due to the increasing antimicrobial resistance. The unavailability of effective antibiotics highlights the need to discover effective alternatives to combat bacterial infections. One option is the use of metallic nanoparticles, which are toxic to some microorganisms due to their nanometric size.

**Methods:**

In this study we (1) synthesize and characterize bismuth and silver nanoparticles, (2) evaluate the antibacterial activity of NPs against *Staphylococcus aureus* and *Escherichia coli* in several infection models (*in vivo* models: infected wound and sepsis and *in vitro* model: mastitis), and we (3) determine the cytotoxic effect on several cell lines representative of the skin tissue.

**Results and discussion:**

We obtained bimetallic nanoparticles of bismuth and silver in a stable aqueous solution from a single reaction by chemical synthesis. These nanoparticles show antibacterial activity on *S. aureus* and *E. coli in vitro* without cytotoxic effects on fibroblast, endothelial vascular, and mammary epithelium cell lines. In an infected-wound mice model, antibacterial effect was observed, without effect on *in vitro* mastitis and sepsis models.

## Introduction

The emergence of multi-drug-resistant bacteria is one of the main concerns in health public sector worldwide. The conventional treatment and prophylaxis strategy against microbial infections is the use of antibiotics; (Schrader et al., 2020) however, these are failing due to the increasing antimicrobial resistance (AMR) (Freeland et al., 2021). It is estimated that by 2050, 4.73 million lives will be lost annually due to infections caused by AMR microorganisms, leading to a predicted global economic impact as high as US$100 trillion (O’Neill, 2015).

Skin wounds are common sites of bacterial infection, impacting human and animal health (Wolcott et al., 2010; Neopane et al., 2018). The most prevalent bacterial strains isolated from infected wounds are *Staphylococcus aureus* and *Escherichia coli* (Călina et al., 2016; Liu et al., 2018; Vasile et al., 2020). These infections can be uncontrollable and invade other tissues and the body, causing life-threatening infections such as sepsis (Mora-Rillo et al., 2015; Paulsen et al., 2015). Moreover, these strains are the most common etiological agents of other infectious processes, such as bovine mastitis (Kateete et al., 2013; Boireau et al., 2018; Saidi et al., 2019). It has been reported that multi-drug resistant bacteria strains can cause this infectious process (Yang et al., 2018; Salauddin et al., 2020).

The unavailability of effective antibiotics highlights the crucial necessity to discover alternatives that could be effective in combating AMR (Uchil et al., 2014). An alternative is the use of metallic nanoparticles (NPs), which can be toxic to some bacteria due to nanometric size between 1 and 100 nm (Leid et al., 2012). NPs can create pores on the bacterial cell wall and thus disorganize, damage, and increase the permeability of the cell membrane (Bilal et al., 2017). Additionally, NPs can locally alter the microenvironment surrounding the bacteria and generate reactive oxygen species (ROS), resulting in cell death (Bilal et al., 2017).

The silver nanoparticles (AgNPs) are the most studied metallic NPs due to their excellent bacteriostatic and bactericidal effects, even on AMR bacteria such as methicillin-resistant *Staphylococcus aureus* (Ansari et al., 2015), which are present in some infected wounds (Li et al., 2023). Other NPs assessed on infected wounds are the bismuth nanoparticles (BiNPs), which are proven to have antimicrobial activity and low cytotoxicity (Shakibaie et al., 2019; da Luz et al., 2020; Vázquez-Munoz et al., 2020; Maliha et al., 2021). Bi_2_O_3_ nanoparticles have been evaluated on 65 strains of methicillin-resistant *Staphylococcus aureus* isolated from hospitalized patients with infected burn wounds and found that these NPs have antimicrobial activity on 16% of isolated strains (Dalvand et al., 2018).

There are reports where the combination of monometallic bismuth and silver nanoparticles has been demonstrated to have antibacterial effects against multi-drug-resistant bacteria. Nonetheless, the combination does not have a synergic effect when administered independently (Iftikhar et al., 2021). In addition, some studies indicated that the use of bimetallic nanoparticles on infected wounds, such as gold and silver (Au-Ag NPs), speeds the healing process, making it more effective and safer, without adverse toxicity (Kumar et al., 2019). We propose that bismuth and silver bimetallic nanoparticles have a greater antimicrobial effect on *S. aureus* and *E. coli* when applied in lower doses than those reported as monometallic nanoparticles, having lower toxicity in eukaryotic cells.

Therefore, we aimed to (1) synthesize and characterize bismuth and silver nanoparticles (Bi/Ag NPs), (2) evaluate the antibacterial activity of these NPs against *Staphylococcus aureus* and *Escherichia coli* in infection models (*in vivo* models: infected wound and sepsis and *in vitro* model: mastitis), and (3) determine the cytotoxic effect on fibroblast, endothelial, and epithelial cell lines.

## Materials and methods

### Nanoparticles synthesis

The metallic nanoparticles were prepared by chemical reduction at a molar ratio of Bi_0.8_Ag_0.2_. All reagents used were of analytical grade. For the synthesis, Bi(NO_3_)_3_.5H_2_O (catalog number NBP100 from Chemika reagents) 3 mM and AgNO_3_ (catalog number NIPL50 from Chemika reagents) 1 mM solutions were used as metallic precursors, and ascorbic acid (1% w/v; catalog number A7506 from Sigma, St Louis, MO, United States) was used as a reducing agent. In addition, citric acid (10% w/v; catalog number C-0759 from Sigma, St. Louis, MO, USA), tartaric acid (0.6% w/v; catalog number A3125 from Jalmek, San Nicolás de los Garza, México), and medium molecular-weight chitosan (0.5% w/v; catalog number 448877 from Sigma, St. Louis, MO, USA) were added to the previous mixture solution at a disposable polystyrene Petri dish. The reaction in aqueous solution was accomplished by UV irradiation of 900,000 μJ/cm^2^ for 20 min in a UV Crosslinker (model CL-1000 from UVP, Cambridge, UK).

### Characterization of bismuth-silver nanoparticles

The surface plasmon resonance (SPR) of bismuth-silver nanoparticles (Bi/Ag NPs) was analyzed by ultraviolet–visible (UV–VIS) spectroscopy. The measurements were performed in a NanoDrop™ 2000 spectrophotometer (Thermo Fisher Scientific, USA). In disposable polystyrene cuvettes, in the range of 200–700 nm. The dynamic light scattering (DLS) technique was used to determine the average hydrodynamic diameter in dilution of 1:10,000 (NPs:water); the electric charge and zeta potential were established using a ZetaSizer Nano ZS90 instrument (Malvern Instruments, Spain). The individual size of metallic nanoparticles was evaluated by a histogram of size distribution from measurements of 100 Bi/Ag NPs from images obtained by scanning electronic microscopy (SEM) with 100,000 magnifications. Nanoparticle shape was determined by transmission electronic microscopy (TEM) with 500,000 magnifications. Nanoparticle elemental composition was determined with a TEM device equipping, an energy-dispersive X-ray spectroscopy (EDS) apparatus, and the compositional analysis was carried out using X-ray photoelectron spectroscopy (XPS) (ULTRA DLD, Shimadzu Ltd., Kyoto, Japan).

### Antimicrobial susceptibility testing

The minimum inhibitory concentration (MIC) and minimum bactericidal concentration (MBC) of Bi/Ag NPs for *Escherichia coli* ATCC 11229 and *Staphylococcus aureus* ATCC 29213 were measured by the broth microdilution method, according to Clinical and Laboratory Standards Institute (Clinical and Laboratory Standards Institute, 2013). In brief, a sterile, disposable, polystyrene, curved-bottom 96-well plate was inoculated with 10^5^ CFU/mL of each of the bacterial strains in Mueller–Hinton broth with different concentrations of Bi/Ag NPs (0.22, 0.43, 0.86, 1.2, 1.5, 1.72, 2.3, 2.9, 3.44, 6.88, 13.76, 27.52, and 55.05 μg/mL). The plate was then incubated at 37°C for 24 h. The optical density was measured at 600 nm wavelength. The MIC was considered as the concentration at which no bacterial growth was observed. The inoculum with the same number of bacteria, without Bi/Ag NPs but treated with gentamicin (10 μg/mL), was used as the control to inhibit the growth of microorganisms. This assay was performed in triplicate. For the MBC assay, from each Bi/Ag NP concentration where no apparent bacterial growth was observed (based on turbidimetry), an aliquot (10 μL) was taken to inoculate in a Petri dish with Mueller–Hinton agar. The plate was incubated at 37°C for 24 h, and the concentration in which there was no growth of CFU was taken as MBC.

### Membrane integrity

The membrane integrity was evaluated by the lactate dehydrogenase (LDH) release using CytoTox 96® Non-Radioactive Cytotoxicity Assay (PROMEGA with catalog number: G1780), following the manufacturer’s recommendations. In summary, 10^5^ CFU/mL of each strain (*E. coli* ATCC 11229 and *S. aureus* ATCC 29213) were inoculated separately in a sterile, disposable, polystyrene, curved-bottom 96-well plate with subinhibitory nanoparticle concentrations of Bi/Ag NPs (0.22, 0.43, 0.86, and 1.72 μg/mL for *S. aureus* and 0.22, 0.43, 0.86, 1.72, and 3.44 μg/mL for *E. coli*) and incubated at 37°C for 24 h. Thereafter, the 96-well plates were centrifuged at 250 xg for 4 min, and aliquots of 50 μL of supernatant were transferred from all wells to new 96-well flat-bottom plates. Then, 50 μL of CytoTox 96 reagent was added to each well (the plate was protected from light for 30 min at room temperature). Next, 50 μL of stop solution were added to each well and incubated for 1 h at room temperature. Finally, the LDH release was determined by measuring OD at 490 nm. To determine LDH maximum release, 10 μL of lysis solution provided by the kit was added (9% v/v Triton® X-100) to 10^5^ CFU/mL of each strain (*E. coli* ATCC 11229 and *S. aureus* ATCC 29213), which was grown in Mueller–Hinton broth for 24 h. This measurement was considered 100% LDH release control. Each treatment measure was performed in duplicate.

### Biofilm formation assay

The static microtiter plate method was used for the semiquantitative determination of biofilm formation using *S. aureus* ATCC 29213 by crystal violet staining. The biofilm assay was only performed in *S. aureus* due to its strong biofilm producer, and this characteristic is a virulence factor that is important for the establishment of infection (Ou et al., 2020). On the other hand, *E. coli* is a moderate-weak biofilm producer (Risal et al., 2018). For this assay, *S. aureus* was passaged by streaking on blood agar plate and incubating at 37°C for 24 h two times after thawing. The strain was adjusted to 0.5 MacFarland Scale and diluted to 1:100 (1.5×10^6^ UFC/ml) in tryptic soy broth (TSB) supplemented with 1% of glucose. Then, 100 μL of bacterial dilution were added per well in a 96-well plate (non-treated flat-bottom); subinhibitory concentrations of Bi/Ag NPs (0.22, 0.43, 0.86, and 1.72 μg/mL) were added per well to sterile TSB with 1% of glucose. Then, the plate was incubated for 24 h at 37°C without agitation. After that, the supernatant was removed, and adhered cells were washed twice with sterile distilled water. Next, the formed biofilm was fixed with 200 μL of methanol for 15 min, and then, 200 μL of 0.1% crystal violet was used to stain it for 15 min. Then, wells were washed five to ten times with sterile distilled water to remove the crystal violet excess. Finally, the plate was dried at 60°C, and the dyed biofilm was dissolved in 200 μL of ethanol-acetone (30:70) solution, to measure its OD at 595 nm. An aliquot of TSB with 1% glucose was used as sterilized control, and other aliquots of inoculum of strain with sterile TSB with 1% glucose and gentamicin (10 μg/mL) were used as negative control. The biofilm formation of *S. aureus* in TSB supplemented with 1% glucose was considered as positive control (100% biofilm formation). The Bi/Ag NP biofilm formation inhibition capacity was evaluated on 3 different days, depending on the time the nanoparticles had been synthesized (7, 14, and 48 days), with the goal of settling the time in which the NPs were more effective. Each assay (per day) was performed in duplicate.

### Cell viability assay

The effect of Bi/Ag NPs on cell viability was determined using the Alamar Blue assay. For this, the endothelial (HUVEC with catalog number CRL-1730), fibroblast (NIH/3 T3 with catalog number CRL-1658), and mammary epithelial (MCF7 with catalog number HTB-22) cell lines were acquired from the American Type Culture Collection (ATCC, Manassas, VA, United States). Cell lines were maintained in Dulbecco’s modified Eagle’s medium (DMEM) supplemented with 10% fetal bovine serum (FBS) and 1% antibiotic-antimycotic solution. Cells were grown to 80% confluence at 37°C, with a humidified atmosphere of 5% CO_2_ and 95% air in an incubator. The cells were plated with density cell of 2×10^4^, 1×10^4^, and 5×10^3^ on 96-well, flat-bottom plates and incubated with the aforementioned conditions for 24 h. After incubation, the culture medium was removed, and cells were washed with PBS and then treated with different concentrations of Bi/Ag NPs (1.72 and 3.44 μg/mL), and the treatment was prepared in DMEM medium. The HUVEC cell line was also evaluated at other Bi/Ag NP concentrations (0.22, 0.43, 0.86, 1.72, 3.44, 6.88, 13.76, 19.52, and 27.52 μg/mL). The plate was incubated for 24 h at 37°C and 5% CO_2_ atmosphere. Then, the supernatant was removed, and the cells were washed twice with DMEM medium. The percentage of relative viability was compared with cells from each cell line without NP treatment. For this, 100 μL of DMEM with 20% Alamar Blue (v/v) were added to each well, and the plate was incubated for 2 h at 37°C in a humidified incubator with 5% CO_2_ (protected from light). Finally, fluorescence intensity was measured on a plate reader using excitation and emission wavelengths of 530 and 590 nm, respectively. Each concentration was performed in triplicate. The cell line without treatment was considered to have a relative viability of 100%. Blank subtraction control was included in each plate.

### Animals

6–8-week-old female BALB/c mice were used for *in vivo* experiments. The animals were kept in 12-h light and dark cycles, with water and food *ad libitum*. Five animals were used per experimental group for all models. All experiments were performed according to the Mexican Official Norm of the technical specifications for the production, welfare, and use of laboratory animals (NOM-062-ZOO-1999) and approved by the internal Comité de Ética de Investigación y Bienestar Animal (CEIBA).

### Infected wound model

The murine-infected wound model was performed according to Adibhesami et al. (2017) with slight modifications; mice were anesthetized using an intraperitoneal injection of ketamine (75 mg/kg) and xylazine (15 mg/kg). The dorsal surface was shaved and wiped with 70% ethanol; next, an excision of 5–6 mm diameter was made on the skin to induce the wound. Mice were distributed randomly into four groups: Group 1 as the control of infected wound with *S. aureus* without treatment, Group 2 as the infected wound with *S. aureus* treated with 22.02 μg Bi/Ag NPs, Group 3 as the control of infected wound with *E. coli* without treatment, and Group 4 as the infected wound with *E. coli* treated with 22.02 μg Bi/Ag NPs. Immediately after the wound was made, it was inoculated with 5×10^5^ CFU of each bacterial strain. The bacterial inoculum was maintained for 2 h at the wound site for infection establishment, and then topical treatment with Bi/Ag NPs was started and applied daily for 5 days. The sacrifice of mice was performed on the 7th day after infection, and the bacterial load at the wound site was determined from the infected wound biopsy. The biopsy was placed in 5 mL of sterile saline solution, and a bacterial suspension was obtained. This suspension was 10-fold serially diluted and plated by spread plate technique in mannitol salt agar for *S. aureus* and EMB plates for *E. coli*, to quantify the bacterial load. Additionally, the biopsies were fixed in 10% buffered formaldehyde and subjected to several steps of histological processing including staining by Masson’s trichrome. The collagen expression was quantified as the percentage of the blue-stained area in the photography using ImageJ software with the color deconvolution function by the FIJI plugin. In addition, groups that are similar to the aforementioned ones were monitored until the wound was healed clinically (Day 10).

### Sepsis model

Mice were separated randomly into three groups: Group 1 as the control, mice without infection only treated with intraperitoneal 22.02 μg Bi/Ag NP injection; Group 2 as mice with sepsis treated with intraperitoneal sterile PBS injection; Group 3 as mice with sepsis treated with intraperitoneal 22.02 μg Bi/Ag NP injection. Mice were kept in polypropylene cages under constant conditions with a temperature of 24°C, 50% relative humidity, and a control light and dark cycle (12 h:12 h). Feed and water were supplied *ad libitum* for 24 h. Group 2 was intraperitoneally injected with 3.6×10^9^ CFU *E. coli* ATCC 11229, and simultaneously sterile PBS was applied. Group 3 was injected intraperitoneally with 3.6×10^9^ CFU *E. coli* ATCC 11229, and 22.02 μg Bi/Ag NPs were applied simultaneously. A second dose of sterile PBS (Group 2) or Bi/Ag NPs (Group 3) was administrated after 2 h of inoculation with *E. coli*. The mice were monitored for 24 h. The sepsis model is induced with *E. coli* because this model has been reported to be highly reproducible in small mammals and displays many features of human sepsis (Poli-de-Figueiredo et al., 2008).

### *In vitro* mastitis model

Bovine raw milk was obtained from the dairy farm of Facultad de Agronomía of the Universidad Autónoma de Nuevo León. The milk sample was transported on ice to the laboratory and kept at 4°C until the assay was performed. In total, 10 mL of milk per flask were inoculated with 1.5×10^5^ CFU/ml of *S. aureus* ATCC 29213 and treated with Bi/Ag NPs at concentrations of 1.72 or 3.44 μg/mL Bi/Ag NPs, 100 μg/mL of gentamicin were used as positive control of inhibition and untreated milk was used as negative control. The milk was maintained for 24 h at 37°C, and the average bacterial load was determined by the spread plate technique of 10-fold serial dilutions in nutritive and mannitol salt agar. This assay was only evaluated in *S. aureus* infection, as this one is the main pathogen causing bovine mastitis (Heikkilä et al., 2018).

### Statistical analysis

The data of *membrane integrity* and *cell viability* assays were analyzed using an ordinary one-way ANOVA test and Tukey’s multiple comparisons between means. For the *Biofilm formation assay,* the data were analyzed by the two-way ANOVA test, and the comparison between means was carried out using Tukey’s multiple comparisons. The data of collagen expression were analyzed by Welch’s *t*-test. The means were considered significantly different with a value of *p* < 0.05. All statistical analyses were performed using GraphPad Prism 8 Software (San Diego, CA, United States).

## Results

### Characterization of bismuth-silver nanoparticles

**Figure 1:**
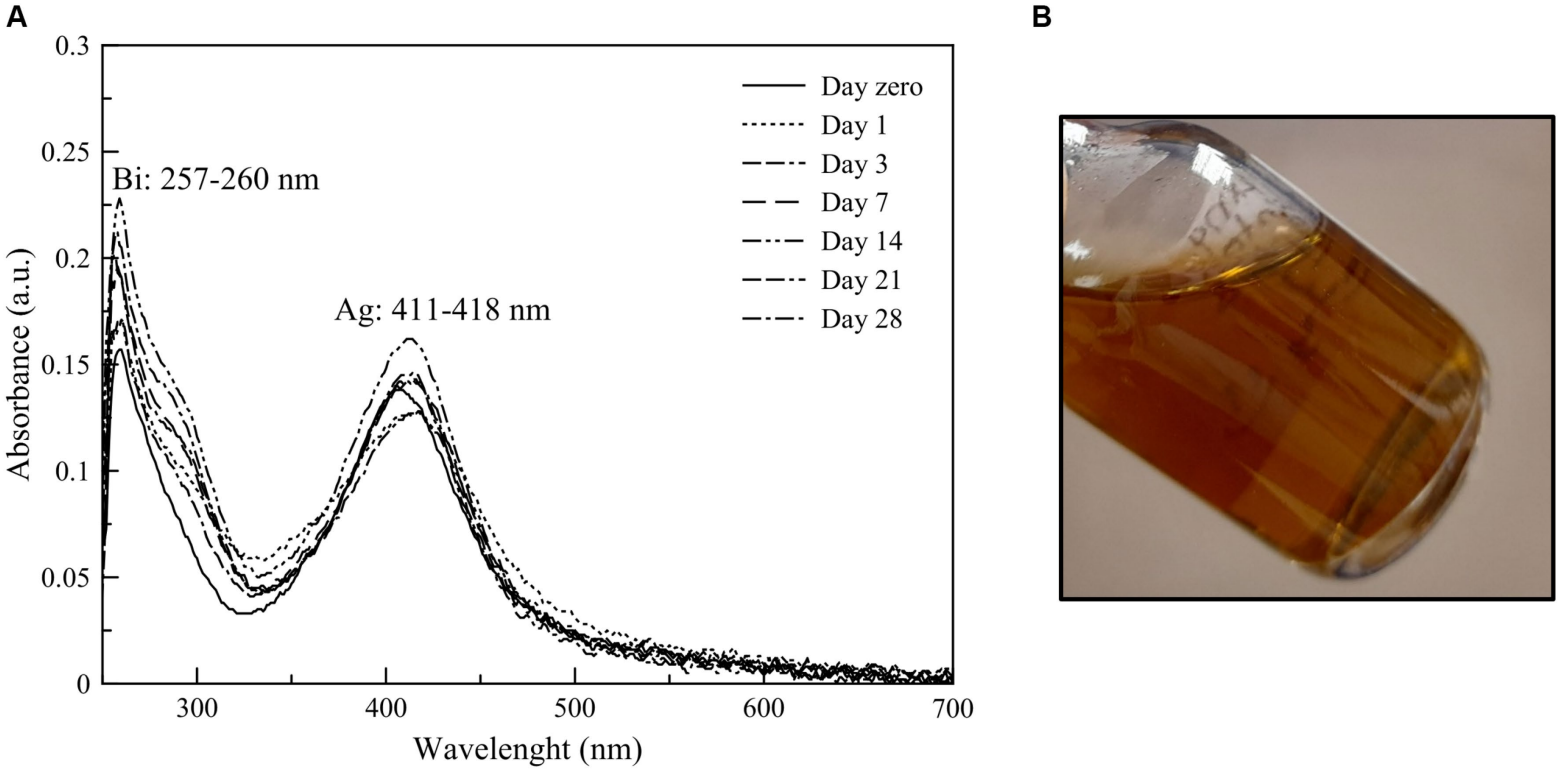
**(A)** Stability of Bi/Ag NPs along 28  days by UV–VIS absorption spectrum and **(B)** Image of Bi_0.8_Ag_0.2_ nanoparticles.

Bismuth and silver nanoparticles were obtained from a single reaction in aqueous synthesis by a chemical method. The SPR of these nanoparticles was characterized by UV–VIS spectroscopy and showed maximum absorbance bands at 257 nm and 411 nm wavelengths. The nanoparticles were stable for at least 28 days after their synthesis (Figure 1).

We then determined the average hydrodynamic size using the DLS technique: 10.15 nm for Bi NPs and 8.06 nm for Ag NPs. The zeta potential was +39.3 and + 39.4 mV for the Bi and Ag NPs, respectively, and the polydispersity index was 0.370 and 0.357 for the Bi and Ag NPs, respectively, (Table 1 along 21 days) achieving stability on 7th day after synthesis.

**Table 1:** Physicochemical parameters (average hydrodynamic diameter, polydispersity index, and zeta potential) of nanoparticles along 21 days.

Days after synthesis	Nanoparticles	Average Hydrodinamic Diameter (nm)	Polidispersity Index (PDI)	Zeta Potential (mV)
Day 0	Bi	242.5	0.529	39.3
	Ag	162.5	0.583	42.2
Day 1	Bi	45.26	0.424	39.2
	Ag	15.74	0.410	39.6
Day 4	Bi	72.31	0.234	38.8
	Ag	3.4	0.328	37.8
**Day 7**	**Bi**	**10.15**	**0.370**	**39.3**
	**Ag**	**8.061**	**0.357**	**39.4**
Day 14	Bi	10.29	0.470	36.0
	Ag	9.69	0.438	36.7
Day 21	Bi	8.553	0.479	33.9
	Ag	13.64	0.410	32.0

**Figure 2:**
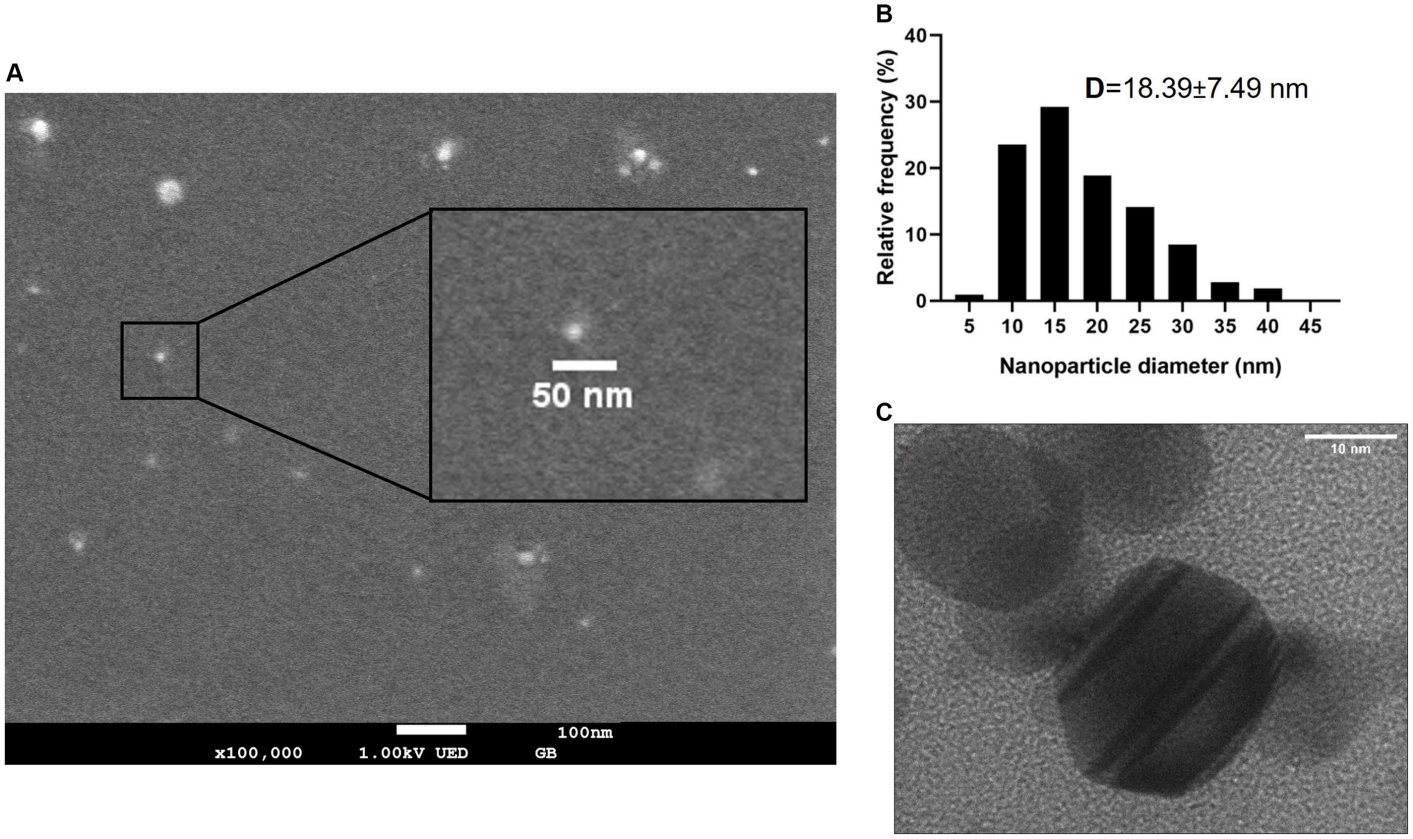
**(A)** Scanning electron microscope (SEM) image of synthesized Bi/Ag NPs. **(B)** Size distribution of Bi and Ag nanoparticles determined by SEM. D: average size and standard deviation of nanoparticles. **(C)** TEM images of synthesized quasi-spheric bismuth and silver nanoparticles with an average size of 18.39 nm.

The average individual size was determined by scanning electron microscopy (SEM) with 100,000 magnifications; a histogram of size distribution was built using ImageJ software version 1.53 from the measurement of 100 Bi/Ag NPs 7 days after synthesis (when the Bi/Ag NPs achieve stability, as shown in Table 1), obtaining an average size of 18.39 ± 7.49 nm. Through the TEM analysis, we determined that Bi/Ag NPs were quasi-spherical (Figure 2).

**Figure 3:**
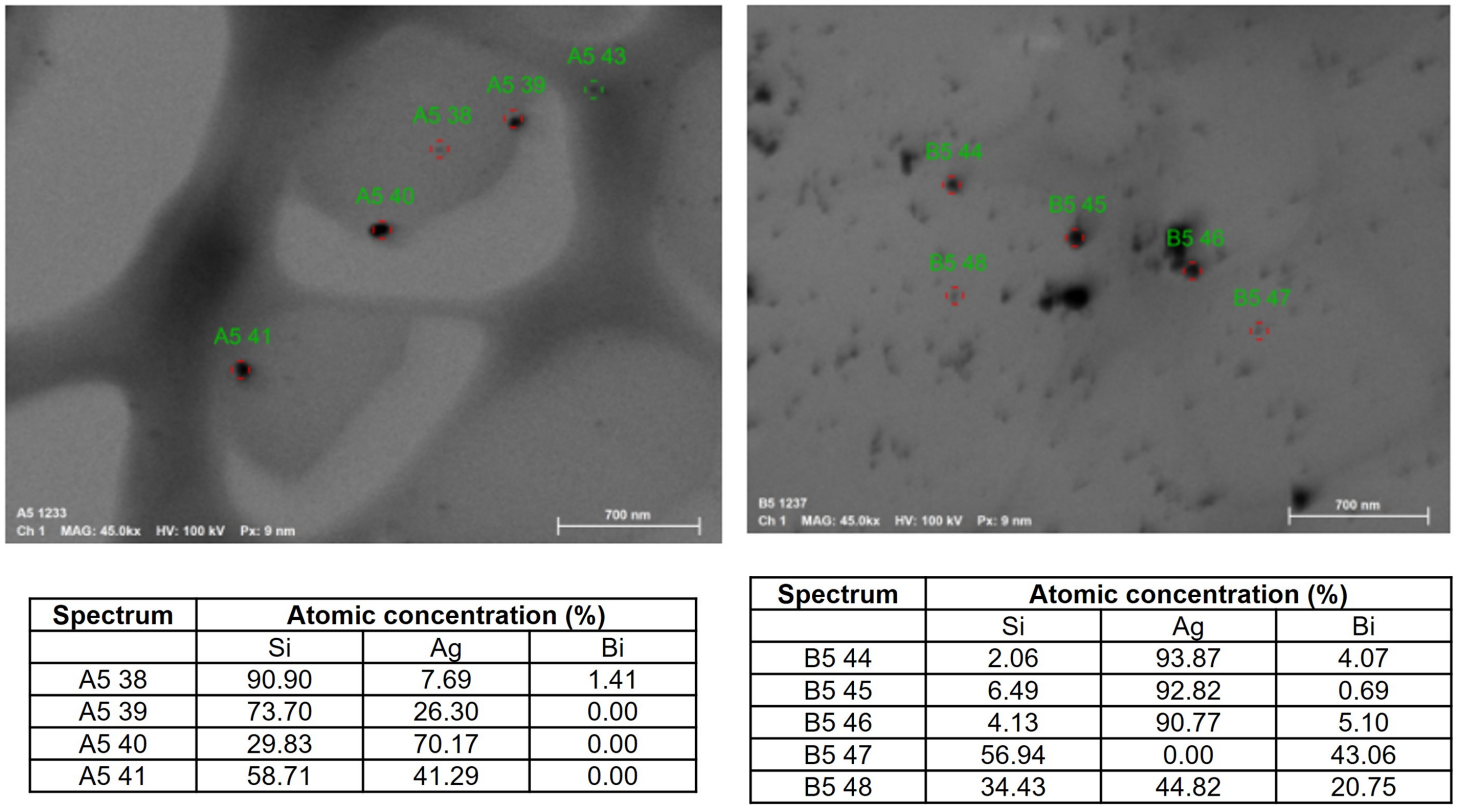
Atomic concentrations determined by elemental analysis of synthesized nanoparticles. The presence of three different populations of nanoparticles was determined in the solution: 11% exist NP monometallic of silver, 33.4% exist NP monometallic of bismuth, and 55.6% correspond to bimetallic NPs of bismuth and silver.

**Figure 4:**
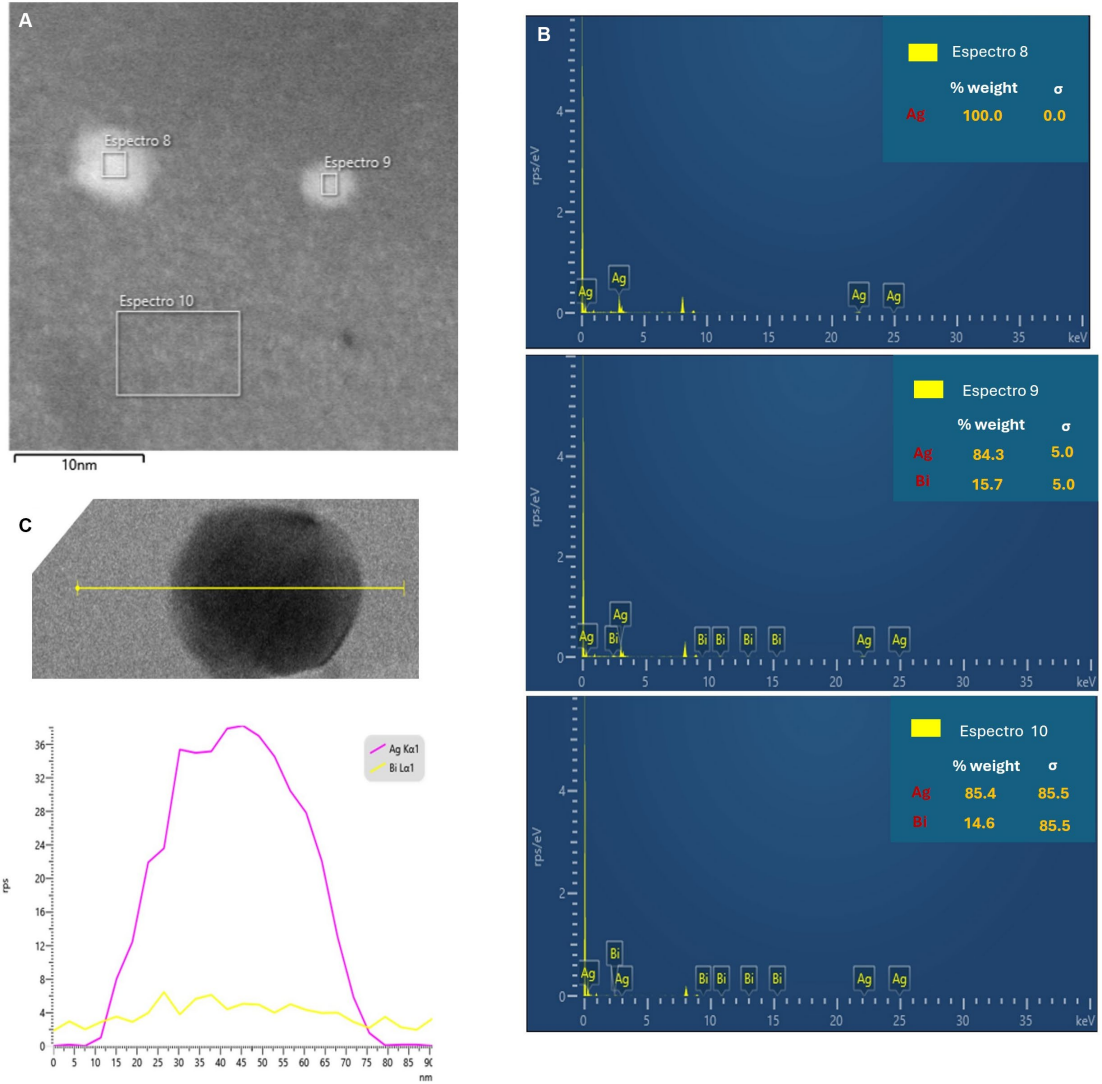
**(A)** Scanning transmission electron microscopy dark field and **(B)** EDS spectrum on the cross-section selection. **(C)** Intensity line profile extracted from the region in yellow line in the elemental maps obtained used EDS.

**Figure 5:**
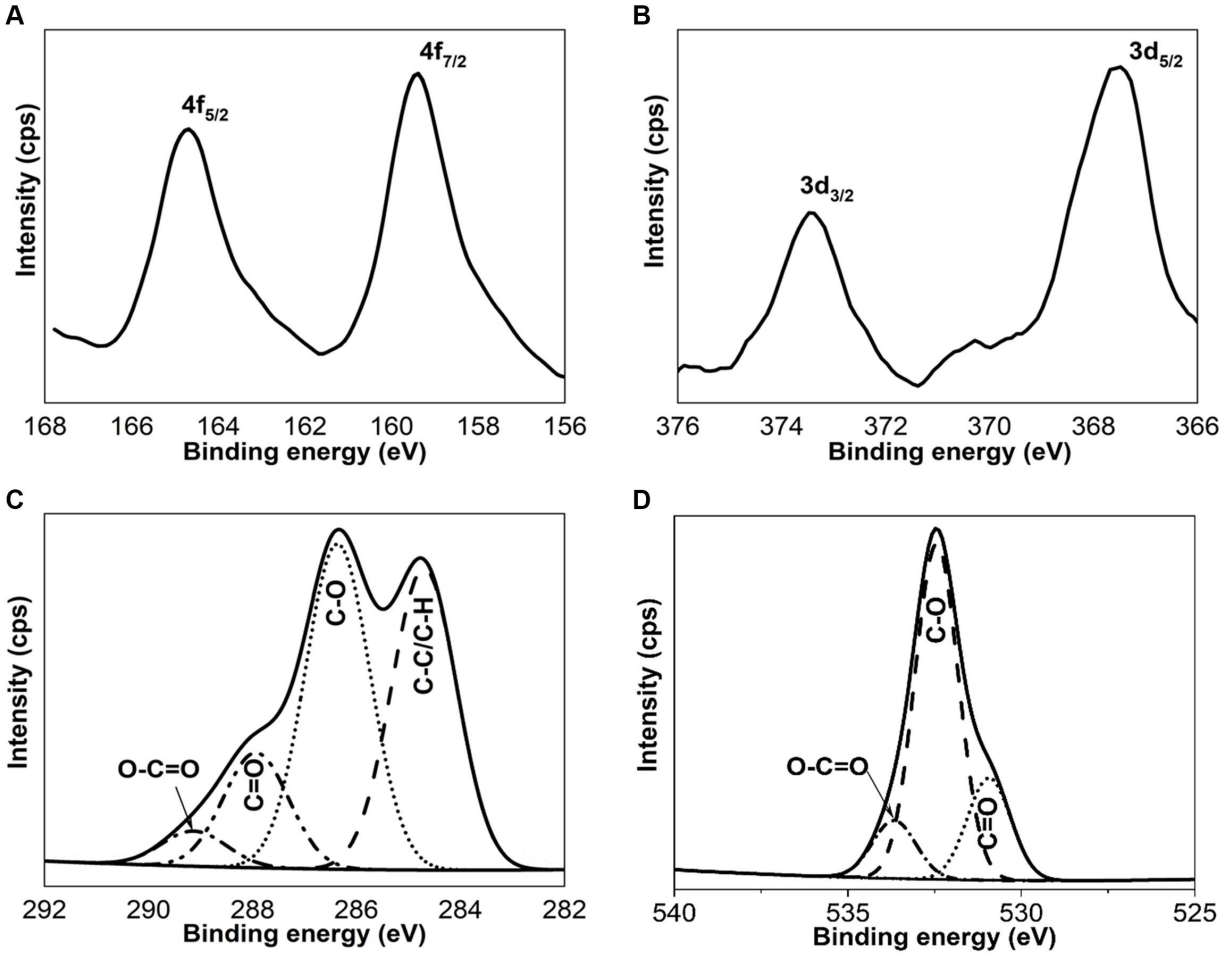
XPS spectra of Bi4f **(A)**, Ag3d **(B)**, C1s **(C)**, and O1s **(D)** of Bi/Ag nanoparticles.

The elemental analysis of Bi/Ag NPs was performed to determine atomic concentrations. In the Bi/Ag NPs synthesis, three different populations were obtained: silver (11%), bismuth (33.4%), and bimetallic bismuth and silver NPs (55.6%) (Figure 3). To confirm the existence of bimetallic nanoparticles, the EDS spectrum was measured by TEM, which was also used to determine the intensity line profile extracted from individual bimetallic NPs (Figure 4). The chemical state of bismuth and silver present in the nanoparticles was determined by XPS. The XPS survey exhibits the presence of Bi, Ag, C, and O (Figure 5). High-resolution XPS spectra at the Bi(4f) electron code level showed two asymmetrical peaks at 164.58 and 159.48 eV corresponding to Bi(4f_5/2_) and Bi(4f_7/2_), respectively. The separation between the peaks of Bi(4f) regions was 5.1 eV, which was consistent with the Bi_2_O_3_, and the presence of not metallic bismuth was observed since metallic bismuth is characterized by a separation between peaks of 5.3 eV and the presence of a peak in the Bi4f region of 157 eV (Ling et al., 2010; Sun et al., 2017). In the case of the Ag(3d), the presence of silver metallic (Δ = 6.0 eV) was confirmed.

### Antimicrobial susceptibility testing

**Figure 6:**
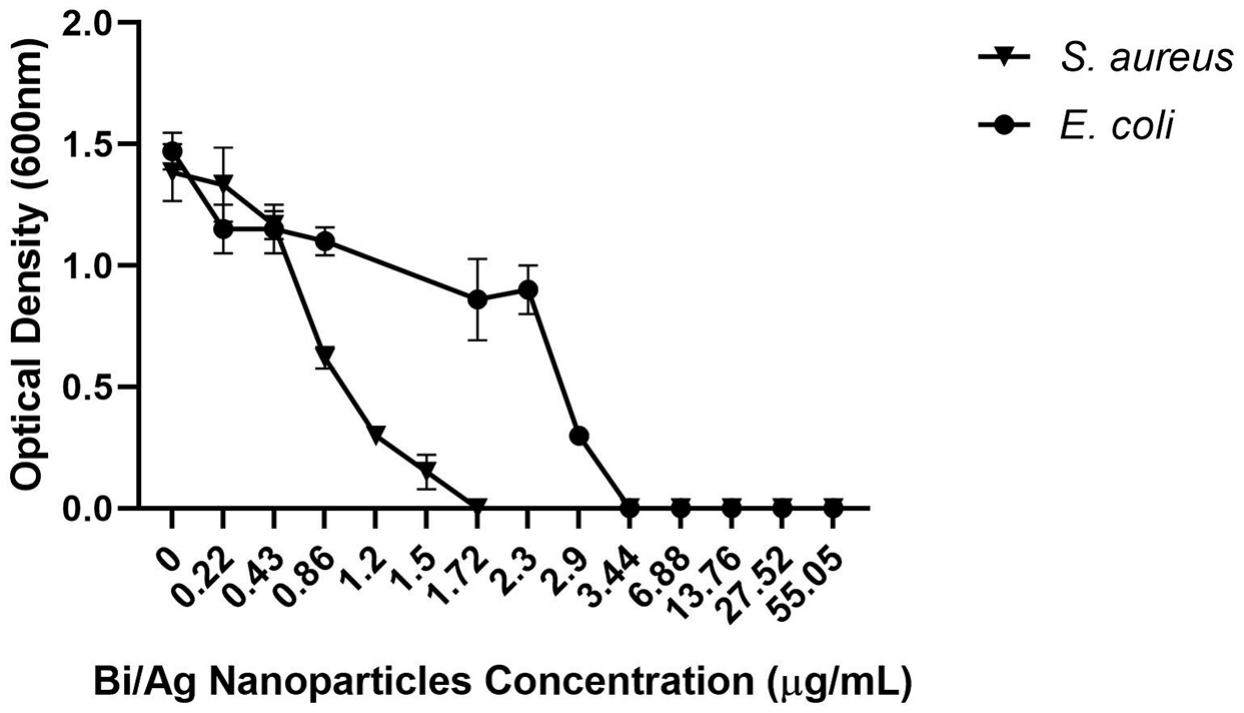
Effect of bismuth/silver nanoparticles on bacterial growth. 10^5^ CFU/mL (*Staphylococcus aureus*, *Escherichia coli*) were treated with bismuth and silver nanoparticles (0.22, 0.43, 0.88, 1.2, 1.5, 1.72, 2.3, 2.9, 3.44, 6.88, 13.76, 27.52, and 55.05 μg/mL) and incubated at 37°C for 24 h. Thereafter, the OD at 600 nm was determined.

In addition, the Bi/Ag NPs have an antibacterial effect on *E. coli* ATCC 11229 and *S. aureus* ATCC 29213 (Figure 6) with MIC and MBC of 3.44 for *E. coli* and 1.72 μg/mL for *S. aureus* (Table 2).

**Table 2:** Minimal Inhibitory concentration (MIC) and minimal bactericidal concentration (MBC) of bismuth/silver nanoparticles for each bacterial strain.

Bacterial strains	MIC (μg/mL)	MBC (μg/mL)
*Staphylococcus aureus* ATCC 29213	1.72	1.72
*Escherichia coli* ATCC 11229	3.44	3.44

### Membrane integrity

**Figure 7:**
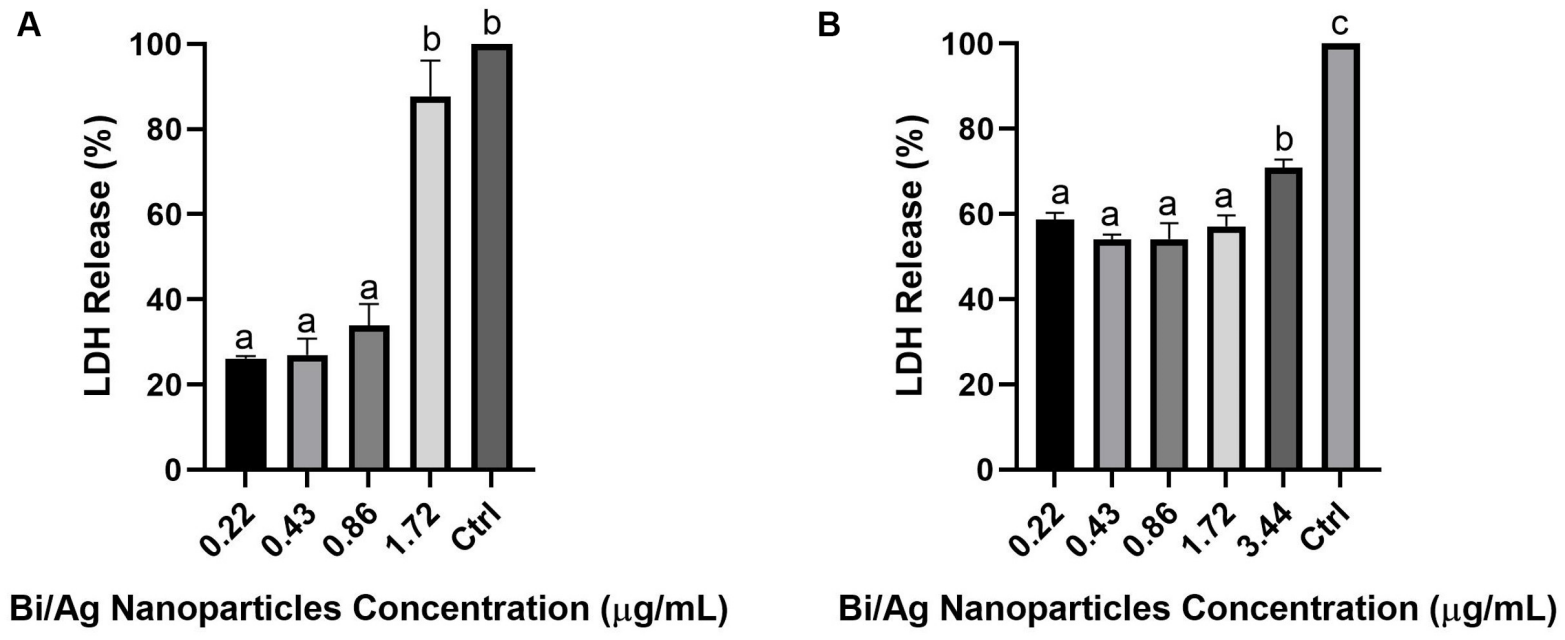
Percentage of LDH release after treatment with bismuth/silver nanoparticle on *Staphylococcus aureus* ATCC29213 **(A)** and *Escherichia coli* ATCC 11229 **(B)**. 10^5^ CFU/mL were inoculated with subinhibitory nanoparticle concentrations (0.22, 0.43, 0.86, 1.72, and 3.44 μg/mL) and incubated at 37°C for 24 h. Thereafter, the LDH release was determined by OD at 490 nm. Each treatment was performed in duplicate. The results represent the average, and the error bars show the standard deviation. Different letters indicate statistically significant difference (*p* < 0.05). Control is 100% of LDH released (cells treated with lysis solution: 9% v/v Triton® X-100).

LDH was released in a dose-dependent manner (*p* < 0.05; Figure 7), indicating that a higher concentration of bismuth and silver nanoparticles increased the rupture of the plasmatic membrane in *E. coli* and *S. aureus*.

### Biofilm formation assay

**Figure 8:**
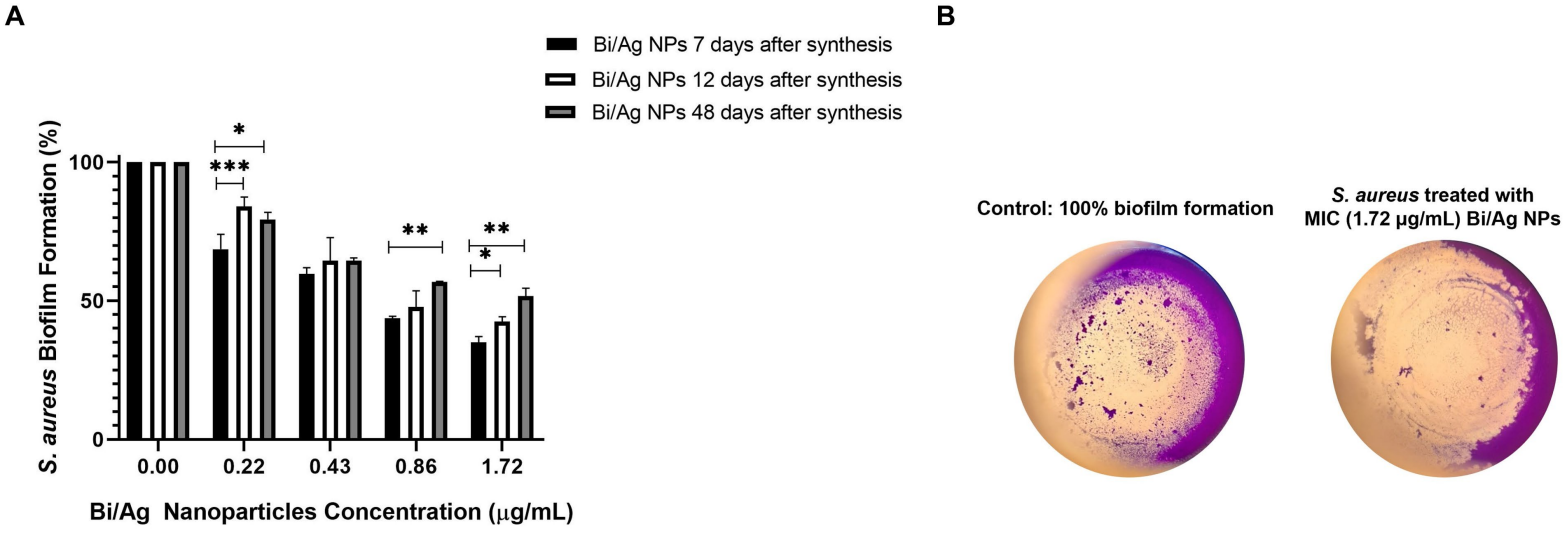
Effect of bismuth/silver nanoparticles after different days of being synthesized tested on inhibition of *Staphylococcus aureus* biofilm formation. 1.5×10^6^ CFU/ml were inoculated and then Bi/Ag NPs were administered (0.22, 0.43, 0.86, and 1.72 μg/mL) and incubated at 37°C for 24 h. Thereafter, the biofilm was stained with violet crystal, and OD was measured at 595 nm for each treatment at least by duplicate. The results represent the average, and the error bars show the standard deviation. ^*^p < 0.05, ^**^p < 0.01, and ^***^p < 0.001 indicate statistical significance between averages **(A)**. Representative images of the biofilm formation assay **(B)**.

The evaluated nanoparticles can inhibit the formation of *S. aureus* biofilm, at higher concentrations, greater the inhibition, but this capacity is decreased in a time-dependent manner (p < 0.05), except for the concentration of 0.43 μg/mL. The Bi/Ag NPs are more effective 7 days after being synthesized (Figure 8).

### Cell viability assay

**Figure 9:**
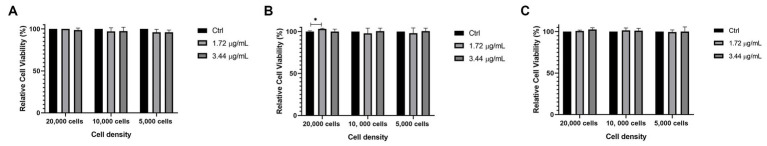
Effect of minimal bactericidal concentration (MBC) of bismuth/silver nanoparticles for *S. aureus* (1.72 μg/mL) and *E. coli* (3.44 μg/mL) on relative viability in HUVEC **(A)**, NIH/3 T3 **(B)**, and MCF7 **(C)** to different cell density (20,000, 10,000, and 5,000 cells) for 24 h. Each treatment was performed in triplicate. The results represent the average, and the error bars show the standard deviation. Cell line without treatment was considered as control. ^*^p < 0.05, ^**^p < 0.01, and ^***^p < 0.001 indicate statistical significance between averages.

**Figure 10:**
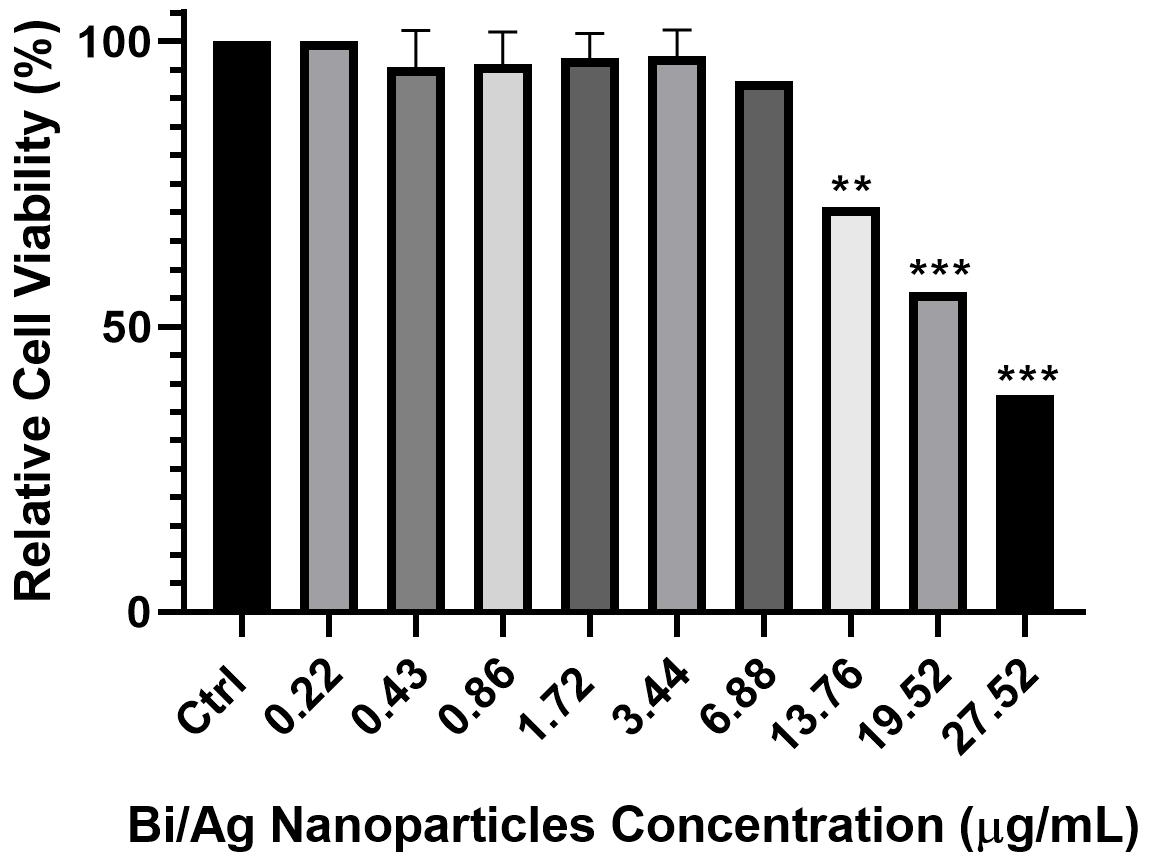
Effect of different concentrations of bismuth/silver nanoparticles on relative viability in HUVEC when exposed to different concentrations (0.22, 0.43, 0.86, 1.72, 3.44, 6.88, 13.76, 19.52, and 27.52 μg/mL) for 24 h. Each treatment was performed in triplicate. The results represent the average, and the error bars show the standard deviation. Cell line without treatment was considered as control. ^*^p < 0.05, ^**^p < 0.01, and ^***^p < 0.001 indicate statistical significance between averages.

The previously established nanoparticle concentrations of MBC for *S. aureus* (1.72 μg/mL) and *E. coli* (3.44 μg/mL) were evaluated in HUVEC, NIH/3 T3, and MCF7 cell lines. It was observed that the viability of these cells was not affected at different cell densities (*p* > 0.05; Figure 9). In addition, to corroborate that nanoparticles did not have any cytotoxic effect on HUVEC cells, a cell viability curve was performed, and it was observed that viability began to decrease from a concentration of 13.76 μg/mL (p < 0.05), eight and four times higher than the MBC used for *S. aureus* and *E. coli*, respectively (Figure 10).

### Infected wound model

**Figure 11:**
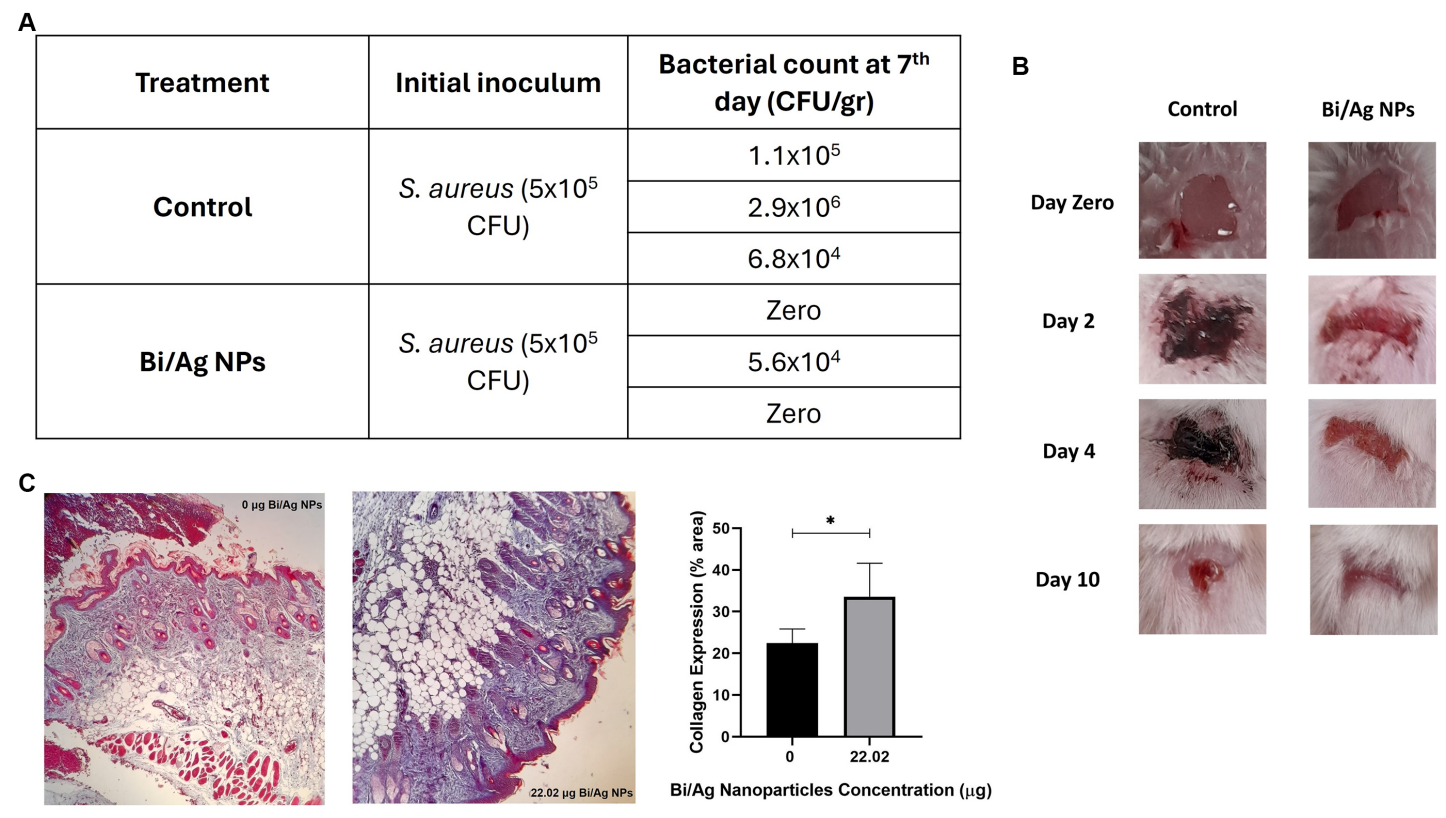
Effect of bismuth/silver nanoparticles on the average bacterial load in wounds contaminated with 5×10^5^ CFU of *S. aureus* in mice. After wound excision, bacteria were inoculated and maintained *in situ* in the wound site for 2 hours to establish infection. Immediately a daily dose of the topical treatment with 22.02 µg Bi/Ag NPs was applied for a period of 5 days. Mice were sacrificed on the 7th day after wound infection, and a biopsy of the wound site was submerged in saline solution to obtain a bacterial suspension, which was further inoculated in salt mannitol agar plates to determine bacterial load **(A)**. Representative images of mice with wounds infected with *S. aureus* and treated with Bi/Ag NPs at zero, 2, 4, and 10 days after of infection establishment **(B)**. Representative images of histological section of mice skin samples, with and without Bi/Ag NPs treatment, stained with Masson’s trichrome (magnification 10X). Red: muscle fiber and hemoglobin; pink: cytoplasm; dark brown or black: cell nuclei and blue: collagen fiber. Data are mean ± standard deviation; ^*^p < 0.05 compared with control, 0 μg Bi/Ag NPs **(C)**.

**Figure 12:**
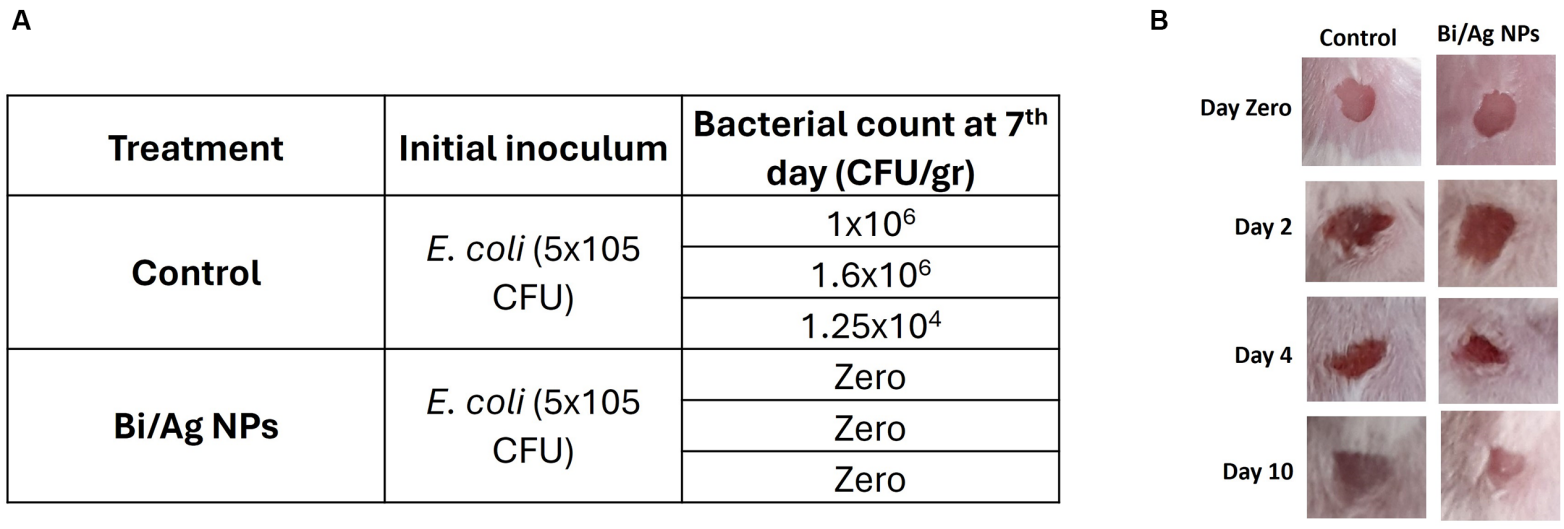
Effect of bismuth/silver nanoparticles on the average bacterial load in wounds contaminated with 5×10^5^ CFU of *E. coli* in mice. After wound excision, bacteria were inoculated and maintained *in situ* in the wound site for 2 hours to establish infection. Immediately a daily dose of the topical treatment with 22.02 µg Bi/Ag NPs was applied for a period of 5 days. Mice were sacrifice on the 7th day after the wound infection, and a biopsy of the wound site was submerged in saline solution to obtain a bacterial suspension, which was further inoculated in EMB agar plates **(A)**. Representative images of mice with wounds infected with *E. coli* and treated with Bi/Ag NPs at zero, 2, 4, and 10 days after infection establishment **(B)**.

An *in vivo* experiment was performed where wounds were infected with inoculums of different strains (*S. aureus* and *E. coli*). The results showed that treatment of 22.02 μg Bi/Ag NPs achieved a reduction in the infection in *S. aureus* (Figure 11) and increased collagen expression (p < 0.05). The treatments also eliminated *E. coli* infection (Figure 12). This way, we demonstrated that the formulation of our nanoparticles proved to be effective in combating and eliminating superficial infections.

### Sepsis model

In this model, the Bi/Ag NPs did not show effectiveness in eliminating this type of infection, given that groups 2 and 3 (sepsis treated and non-treated with Bi/Ag NP) died 24 h after infection, and there was no difference between them. However, group 1 (treated only with Bi/Ag NPs) survived, so the Bi/Ag NPs had no toxic effect.

### *In vitro* mastitis model

The results showed that Bi/Ag NPs did not eliminate the infection in milk, since it was not possible to eliminate the bacteria present in it (Table 3), whereas those were already present in the milk sample or were inoculated.

**Table 3:** Effect of different concentrations of bismuth/silver nanoparticles on average bacterial load in raw milk contaminated with 1.5×10^5^ CFU/ml of *S. aureus ATCC 29213*.

Treatment	Initial inoculum (*S. aureus*, CFU/mL)	Bacterial count in nutritive agar at 24 h after infection	Bacterial count in salt-mannitol agar at 24 h after infection
Control 1	0	Uncountable	Uncountable
Control 2	1.5×10^5^	Uncountable	Uncountable
Bi/Ag NPs (1.72 μg/mL)	1.5×10^5^	Uncountable	Uncountable
Bi/Ag NPs (3.44 μg/mL)	1.5×10^5^	Uncountable	Uncountable
Gentamicin	1.5×10^5^	Zero	Zero

The infected raw milk was maintained for 24 h at 37°C and, the bacterial load was determined by spread plate technique using serial dilutions in nutritive and salt mannitol agar.

## Discussion

The system of nanoparticles developed (Bi/Ag NPs) is composed of three different populations: bimetallic nanoparticles of bismuth and silver and monometallic nanoparticles of either silver or bismuth (Figure 3, the presence of Si is due to the material of the grid where the sample was placed for analysis). The synthesis of bimetallic nanoparticles was possible because bismuth is soluble in silver at 5.5% (Karakaya and Thompson, 1993), and this explains why the reaction efficiency was not 100%, finding monometallic nanoparticles of each element. In addition, the composition distribution of bimetallic NPs is not homogeneous (weight ratio Bi_15.7_Ag_84.3_, Figure 4), despite the initial molar ratio (Bi_0.8_Ag_0.2_). Similar results were observed in a study where bismuth and silver bimetallic nanoparticles were generated by the mechano-chemistry reduction method (Ruiz-Ruiz et al., 2016). Our novelty synthesis permits the obtaining of stable bismuth and silver nanoparticles in an aqueous solution from a single reaction by chemical reduction. In addition, this is the first solution of bimetallic bismuth and silver nanoparticles with antibacterial activity.

Our NP formulation is characterized and showed to be stable because zeta potential is greater than 30 mV (Kovacevic et al., 2011), since this parameter indicates the magnitude of the electrostatic attraction or repulsion force between them which prevents crowding. Also, maximum absorbance bands were observed at 257 and 411 nm wavelengths, characteristic of bismuth surface plasmon resonance (Ruiz-Ruiz et al., 2016; Das et al., 2020), and silver NPs (Piñero et al., 2017), respectively.

The Bi/Ag NPs have different sizes with an individual average size of 18.39 ± 7.49 nm and quasi-spherical shape (Figure 2). This size and shape favor successful entry into the bacteria, inducing bactericidal activity. This might be due to a greater surface area and releasing of a major number of ions with high antimicrobial activity compared with NPs with other morphologies (Cheon et al., 2019).

*S. aureus* was more susceptible to Bi/Ag NPs than *E. coli* (Figure 6). This may be due to the positive electrical charge of the NPs, since the cell wall of gram-positive bacteria is composed of teichoic and lipoteichoic acids that confer them a greater negative electrical charge than gram-negative bacteria (Liu et al., 2015), so there is probably a greater electrostatic attraction between Bi/Ag NPs and gram-positive bacteria. The inhibition of biofilm formation (Figure 8) may be the result of the fact that the NPs are probably able to decrease the transcriptional activity of genes that are responsible for biofilm formation as has been previously reported with silver NPs (Yu et al., 2018). However, one of the disadvantages, when using our Bi/Ag NP synthesis reduced with organic acid (ascorbic acid and citric acid) and stabilized with chitosan, is the decrease in biofilm formation inhibition capacity when used after 48 days of elaboration. This is necessary to develop Bi/Ag NPs using other reductors and stabilizer agents, to corroborate if these factors can maintain the anti-biofilm capability for a long time.

Bismuth and silver monometallic nanoparticles have been evaluated for wound healing. The silver NPs demonstrated to have antibacterial effects on *S. aureus* in infected wounds (Ansari et al., 2015; Li et al., 2023), and several studies showed that these NPs are more effective in combination with antibiotics (Ahmadi and Adibhesami, 2017; Khalil et al., 2021). However, the silver NPs can induce citotoxicity in cells involved in the wound healing process, such as keratinocytes and fibroblasts (Wilkinson and Hardman, 2020; Skóra et al., 2021). Thus, it is necessary to develop new alternatives for combating infectious processes. The Bi_2_O_3_ NPs have been tested to evaluate their effects on the healing of superficial wounds, but without infection, demonstrating accelerated wound healing (Dalvand et al., 2018; Hassan et al., 2023). It is also known that bimetallic nanoparticles have greater antimicrobial activity compared with the use of monometallic nanoparticles (Gulam Mohammed et al., 2014; Perdikaki et al., 2016; Kumar et al., 2019; Arora et al., 2020). Therefore, our formulation of metallic NPs is an ideal candidate to combat infection mainly in superficial wounds because it can eliminate the bacterial load of established infections and does not present toxicity on endothelial, epithelial, and fibroblast cells (Figure 9), which are necessary for angiogenesis and tissue regeneration (Wilkinson and Hardman, 2020), a crucial process for wound healing. Moreover, increased collagen expression may be indicative of improved wound healing (Masson-Meyers et al., 2020) because collagen deposition plays a central role during tissue regeneration, wound remodeling, and wound healing (Zhang et al., 2019; Mathew-Steiner et al., 2021).

The most common gram-positive bacteria that can appear at the wound site is *Staphylococcus aureus* and the most common gram-negative bacteria is *Escherichia coli*; gentamicin was used as control in antimicrobial susceptibility assays, as it is a broad-spectrum aminoglycoside antibiotic with the capacity to bind to the 16 s RNA at the 30S ribosomal subunit, disturbing mRNA translation and leading to the formation of truncated or non-functional proteins. Gentamicin exhibits bactericidal activity against broad-spectrum bacteria, inhibiting its growth, and is a good option to treat several common infections, such as those caused by *Escherichia coli* and *Staphylococcus aureus* (Gemeinder et al., 2021). These microorganisms are capable of colonizing wounds, forming biofilm considered the main virulence factor that affect the correct wound healing process. (Wolcott et al., 2010; Vasile et al., 2020). Our formulation causes the death of microorganisms by the rupture of the plasmatic cell membrane and finally release of the cytosolic content to the extracellular environment as observed in the release of the LDH enzyme assay (Figure 7). In addition, Bi/Ag NPs inhibit biofilm formation and prevent the appearance of chronic infections. Notably, the MBC of Bi/Ag NPs on bacteria studied is up to 100 times lower than those reported by other studies, where the antibacterial effect of bismuth and silver nanoparticles on *in vitro* models was evaluated (Kovacevic et al., 2011; Lange et al., 2021; Jawad et al., 2022).

Our system of Bi/Ag NPs showed antibacterial effects on the infected wound model (Figures 11, 12) but not on sepsis and *in vitro* mastitis models (Table 3). The last two models represent a generalized infection with the presence of severe inflammation and significant physiological imbalance (Bradley, 2002; O’Brien Jr et al., 2007). Nowadays, developing or prescribing treatments to combat this type of infection is a challenge due to the unavailability of effective antibiotics for these pathologies (Dumache et al., 2015; Gonçalves et al., 2022). Hence, we think that it might be necessary to combine our formulation with other products for greater effectiveness in these clinical conditions.

## Conclusion

Based on these results, we concluded that it is possible to obtain bimetallic nanoparticles of bismuth and silver in a stable aqueous solution from a single reaction by chemical synthesis. Even more, this synthesis possesses antibacterial activity on *S. aureus* and *E. coli in vitro* and *in vivo* in an infected wound mice model, without showing cytotoxic effect on fibroblast, endothelial vascular, and mammary epithelium cell lines. We suggest more studies to test this formulation on more bacterial strains and infected wounds to create a commercial application.

## Data availability statement

The raw data supporting the conclusions of this article will be made available by the authors, without undue reservation.

## Ethics statement

The animal study was approved by Comité de Ética de Investigación y Bienestar Animal (CEIBA), Facultad de Ciencias Biológicas, Universidad Autónoma de Nuevo León, San Nicolás de los Garza, Nuevo León, México. The study was conducted in accordance with the local legislation and institutional requirements.

## Author contributions

BC-V: Writing – review & editing, Writing – original draft, Project administration, Methodology, Investigation, Formal analysis, Data curation, Conceptualization. MF-M: Writing – review & editing, Validation, Supervision, Investigation, Funding acquisition, Conceptualization. DZ-T: Writing – review & editing, Validation, Supervision, Methodology. LV-T: Writing – review & editing, Validation, Supervision, Methodology. JK: Writing – review & editing, Supervision. PG-C: Writing – review & editing, Investigation. GS-H: Writing – review & editing, Formal analysis, Data curation. CR-P: Writing – review & editing, Supervision, Resources, Project administration, Investigation, Funding acquisition.
