# Supplementary figures and images for: Antibacterial efficacy of novel bismuth-silver nanoparticles synthesis on Staphylococcus aureus and Escherichia coli infection models
Source: Front Microbiol. 2024 Apr 8;15:1376669. doi: 10.3389/fmicb.2024.1376669 (PMC11033500; doi:10.3389/fmicb.2024.1376669)

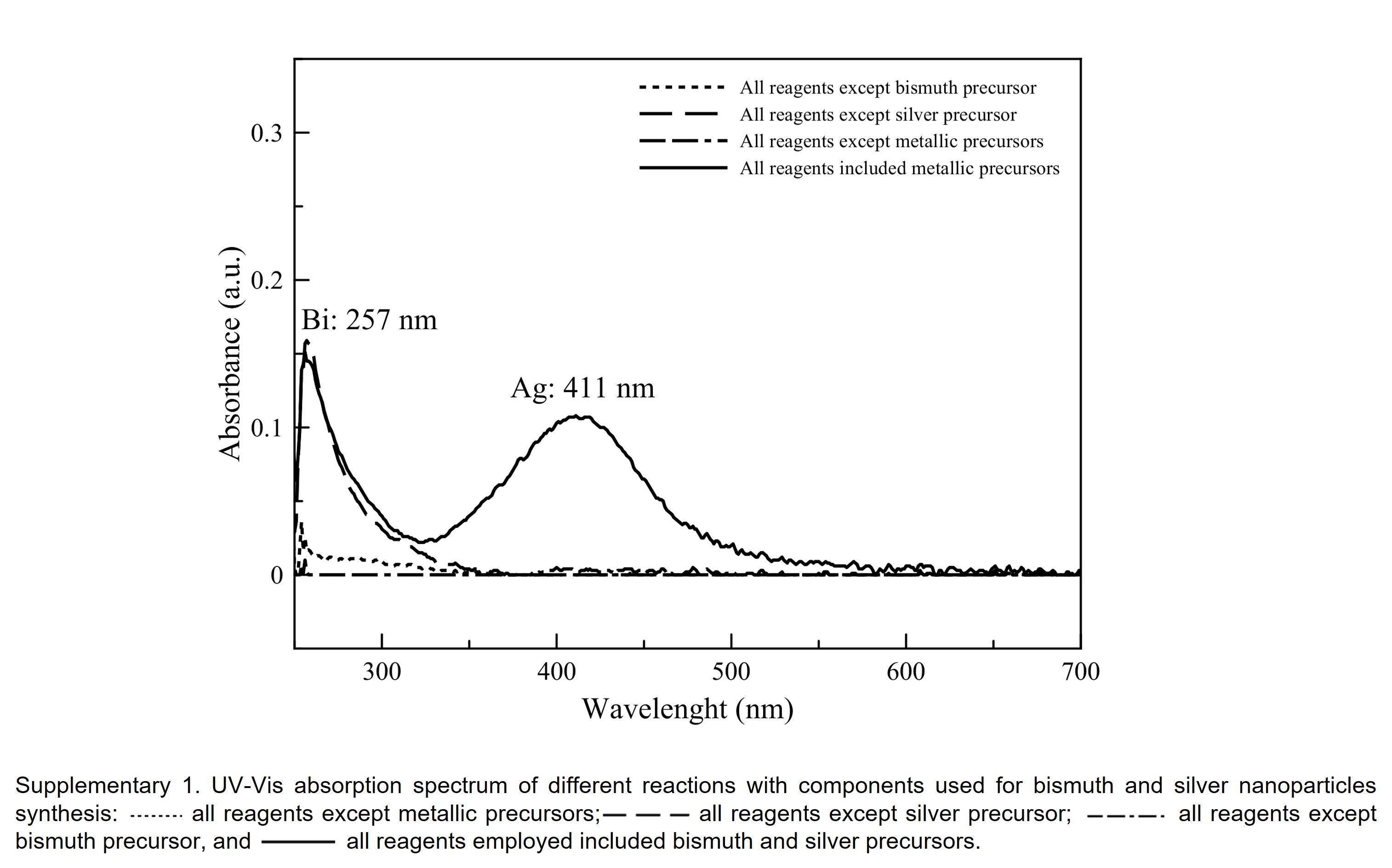

Supplement: Supplementary file 1 [file Image_1.tiff]
